# Supplementary figures and images for: Biological Effects of HDAC Inhibitors Vary with Zinc Binding Group: Differential Effects on Zinc Bioavailability, ROS Production, and R175H p53 Mutant Protein Reactivation
Source: Biomolecules. 2023 Oct 28;13(11):1588. doi: 10.3390/biom13111588 (PMC10669723; doi:10.3390/biom13111588)

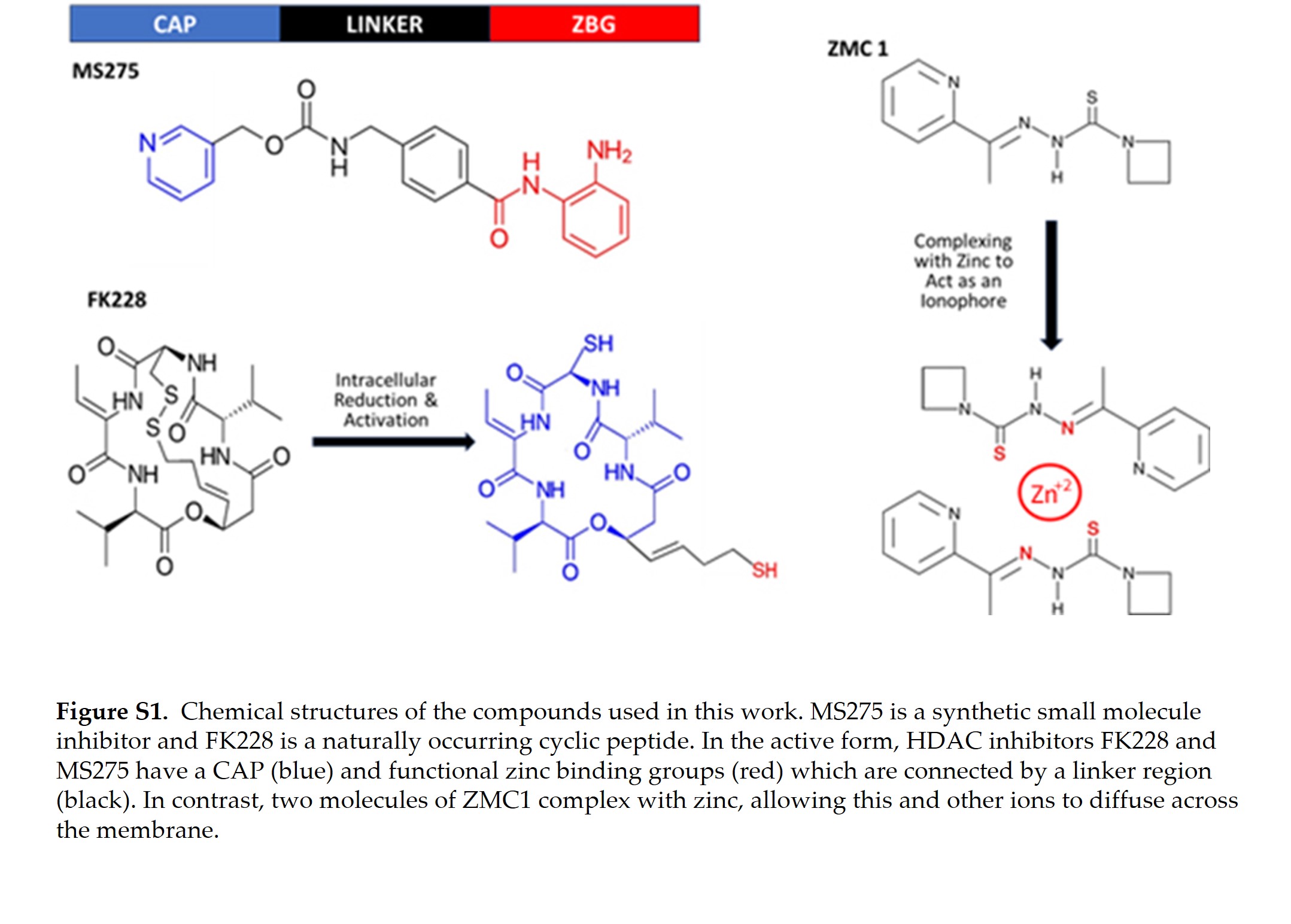

Supplement: Supplementary file 1 [file biomolecules-13-01588-s001.zip › Figure S1.jpg]

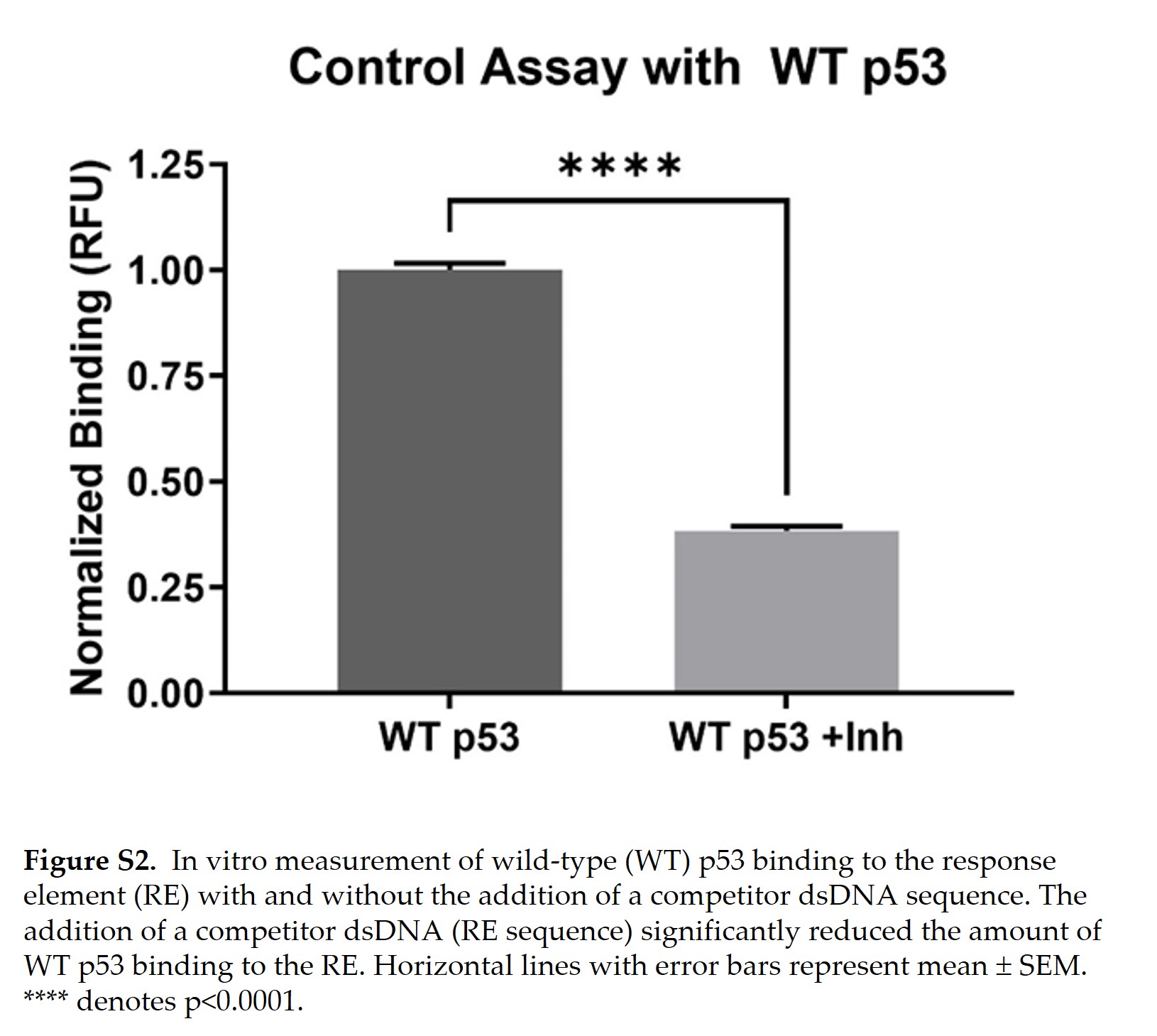

Supplement: Supplementary file 1 [file biomolecules-13-01588-s001.zip › Figure S2.jpg]

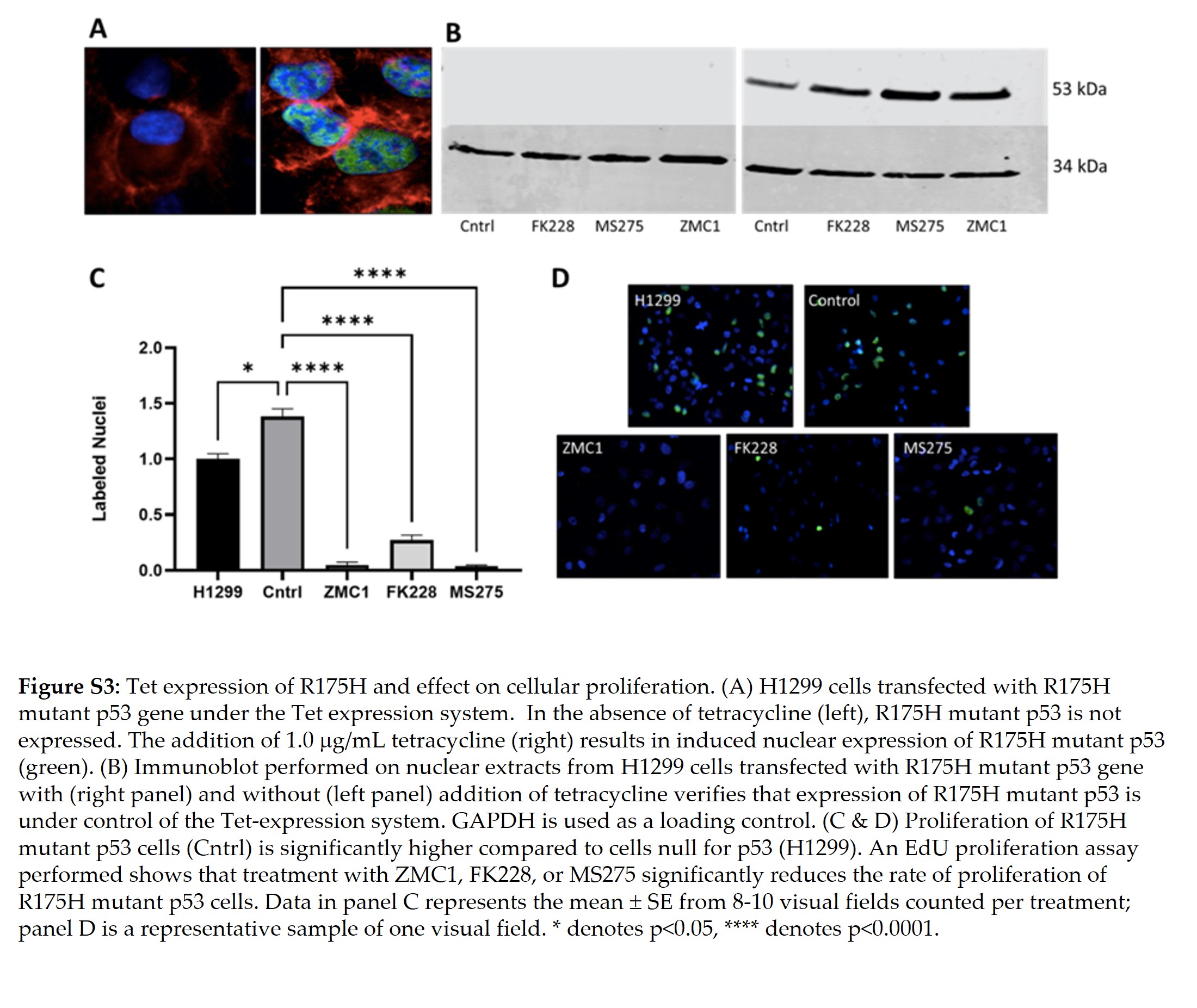

Supplement: Supplementary file 1 [file biomolecules-13-01588-s001.zip › Figure S3.jpg]

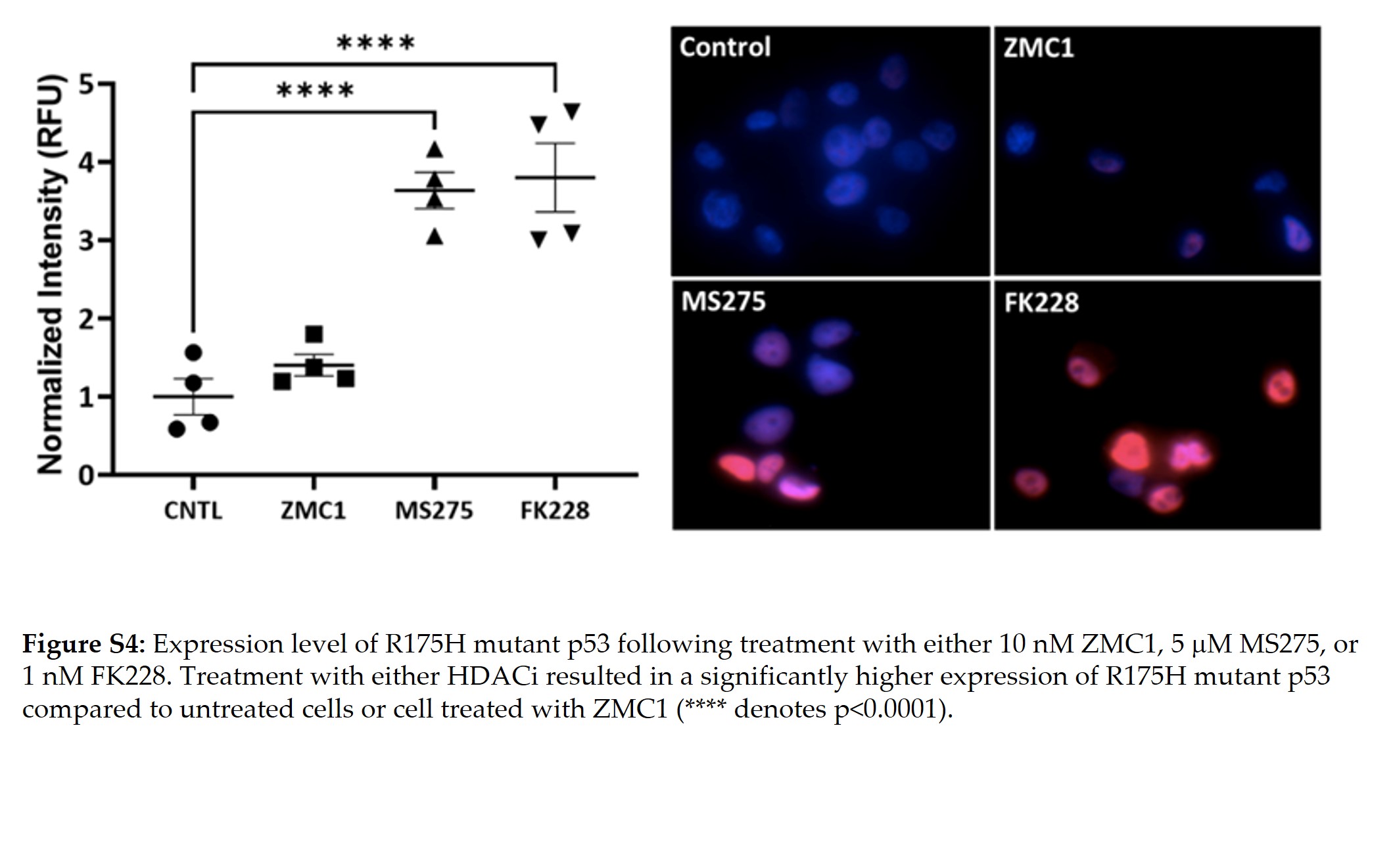

Supplement: Supplementary file 1 [file biomolecules-13-01588-s001.zip › Figure S4.jpg]

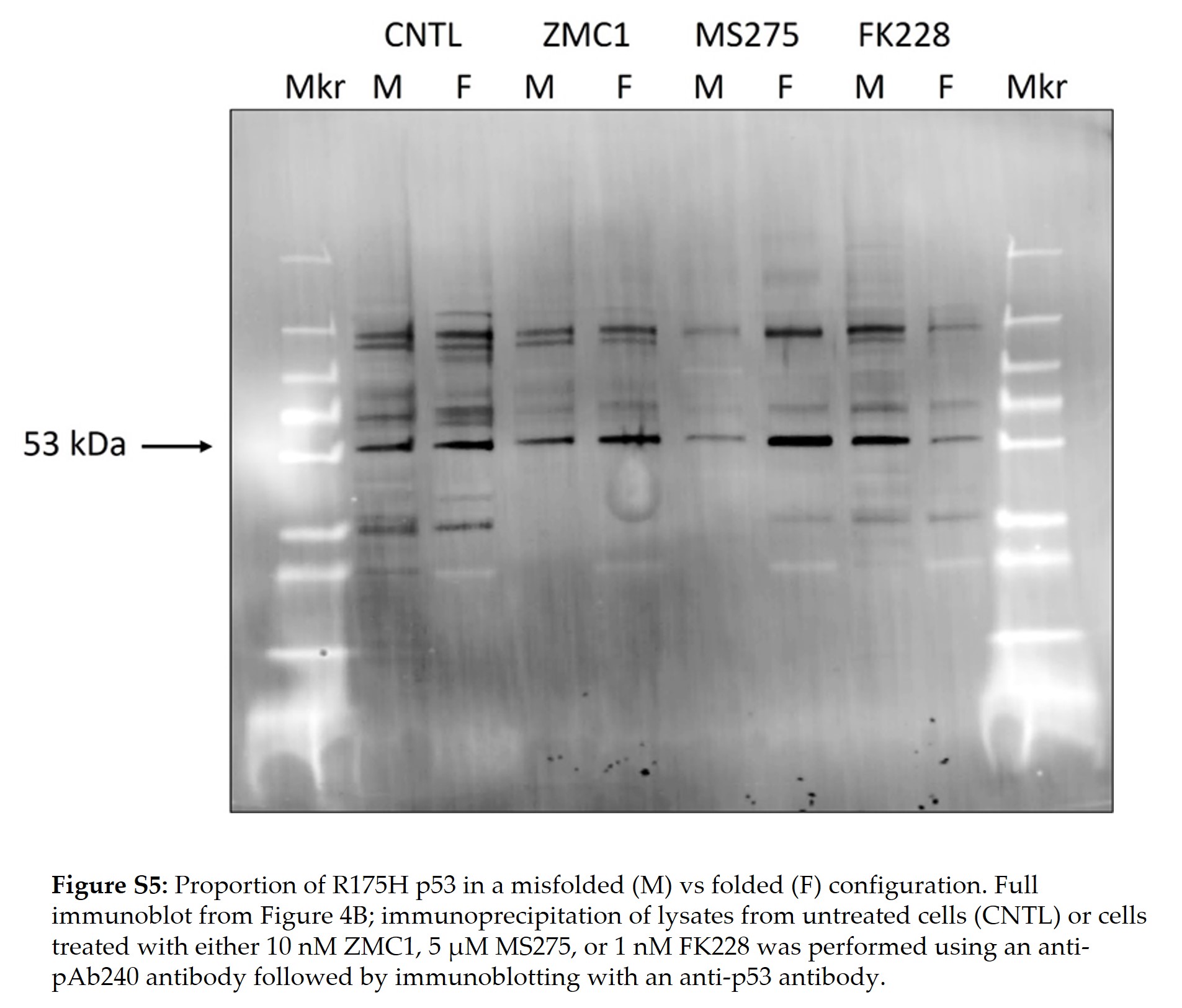

Supplement: Supplementary file 1 [file biomolecules-13-01588-s001.zip › Figure S5.jpg]

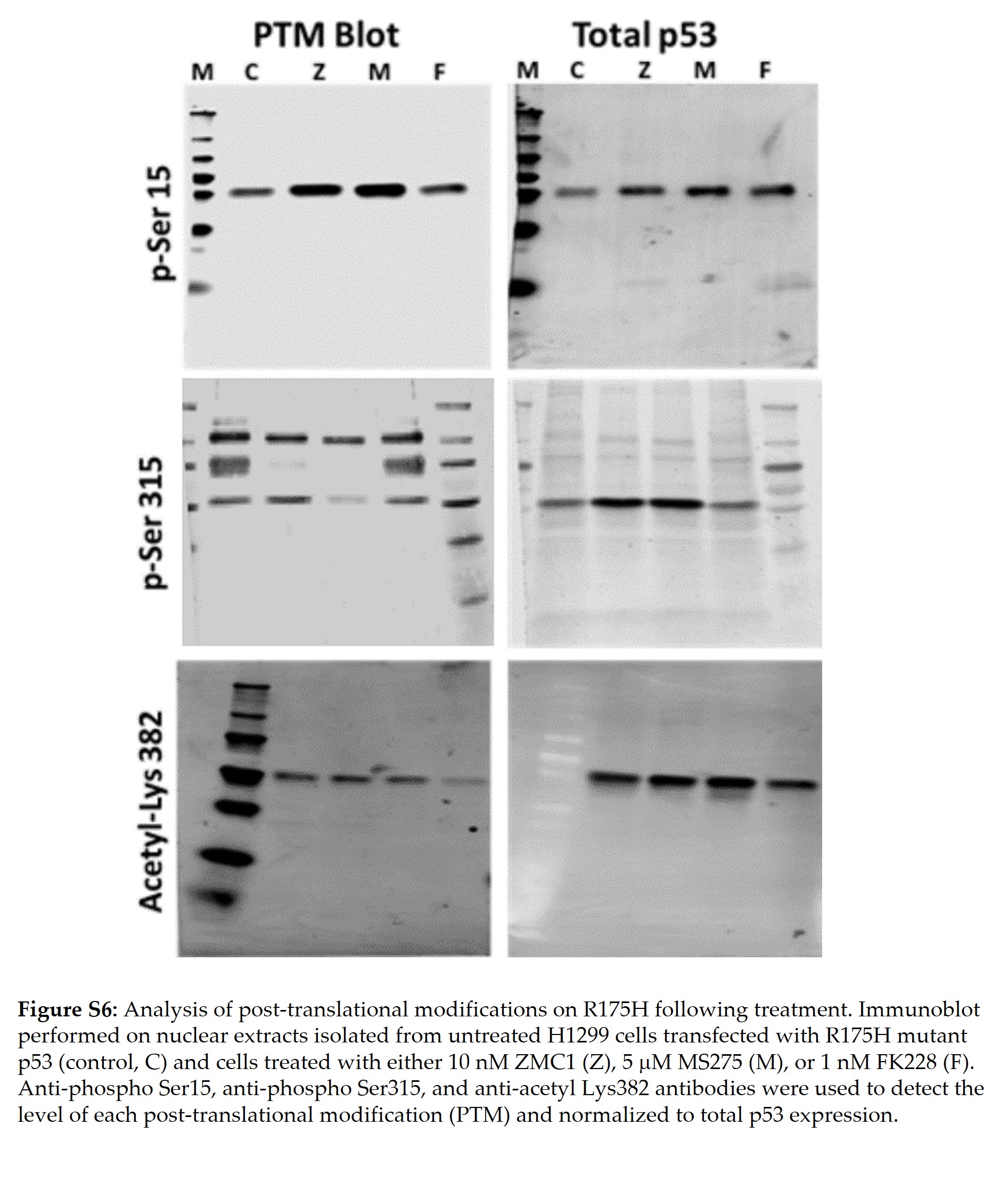

Supplement: Supplementary file 1 [file biomolecules-13-01588-s001.zip › Figure S6.jpg]

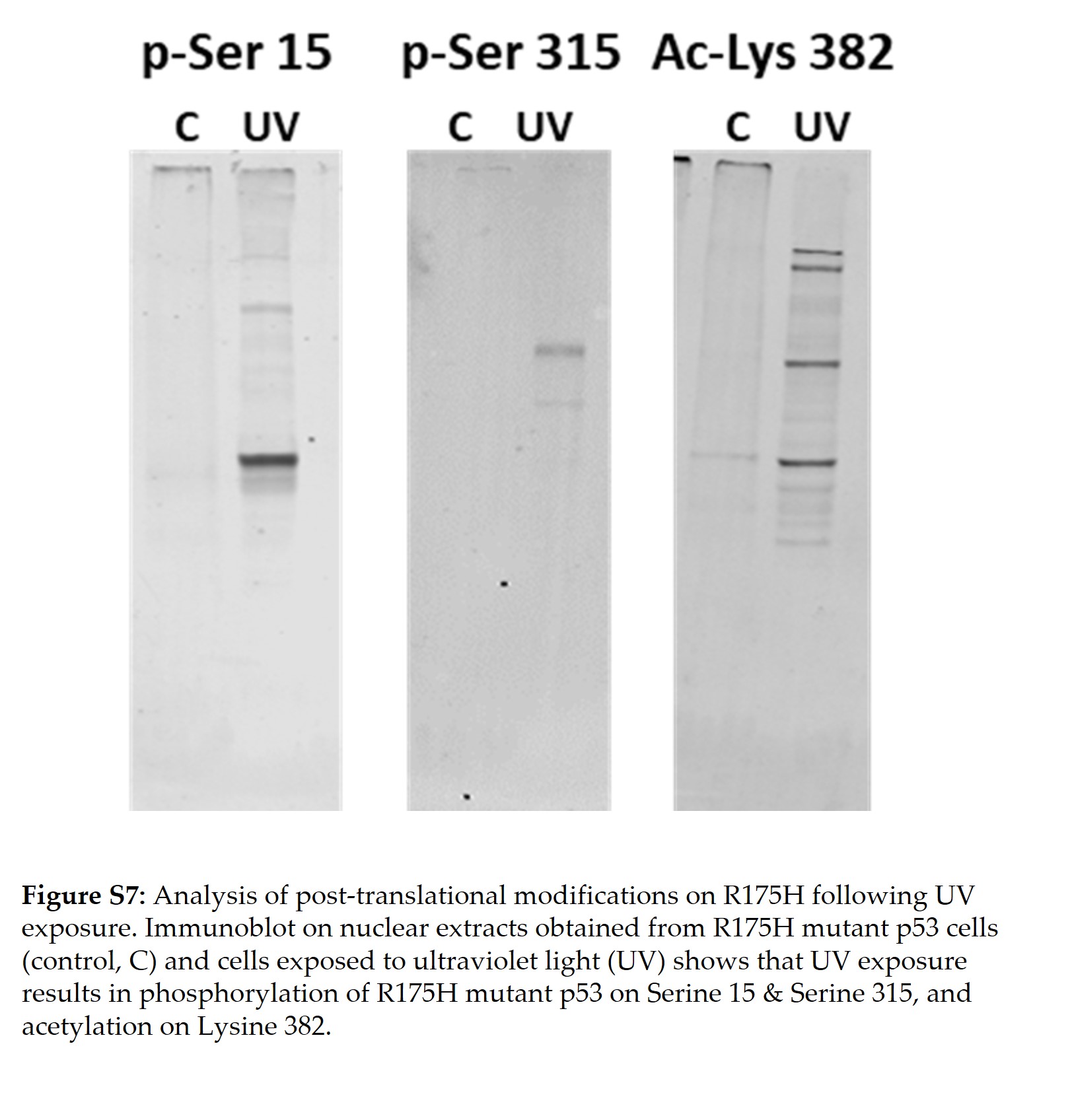

Supplement: Supplementary file 1 [file biomolecules-13-01588-s001.zip › Figure S7.jpg]

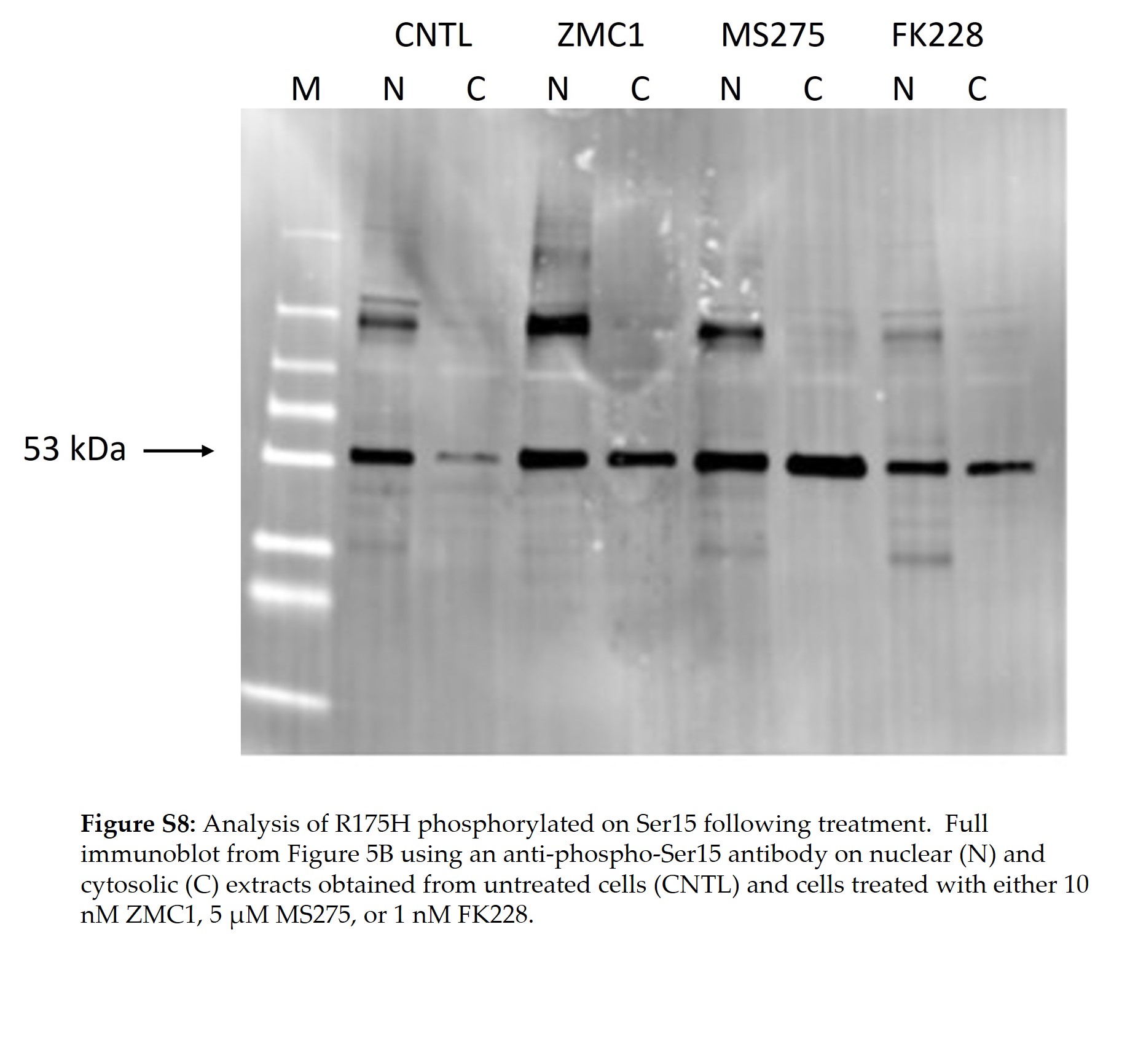

Supplement: Supplementary file 1 [file biomolecules-13-01588-s001.zip › Figure S8.jpg]
